# Supplementary material for: Design of Fragrance Formulations with Antiviral Activity Using Bayesian Optimization
Source: Microorganisms. 2024 Jul 31;12(8):1568. doi: 10.3390/microorganisms12081568 (PMC11356527; doi:10.3390/microorganisms12081568)
Supplement: Supplementary file 1 [file microorganisms-12-01568-s001.zip › microorganisms-3104575-supplementary.pdf]

Design of fragrance formulations with antiviral activity using Bayesian optimization

Fan Zhang[a,b], Yui Hirama[c], Shintaro Onishi[c], Takuya Mori[c],

Naoaki Ono[a,d], Shigehiko Kanay[a,d*]

[a] Division of Information science, Graduate School of Science and Technology, Nara Institute of Science and Technology, 8916-5 Takayama-cho, Ikoma, Nara, 630-0192, Japan.

[b] Material Science Research, Kao Corporation, 1334 Minato, Wakayama-shi, Wakayama, 640-8580, Japan.

[c] Biological Science Research, Kao Corporation, 2606 Akabane, Ichikai-machi, Haga-gun, Tochigi, 321-3426, Japan.

[d] Data Science Center, Graduate School of Science and Technology, Nara Institute of Science and Technology, 8916-5 Takayama-cho, Ikoma, Nara, 630-0192, Japan.

Table S1. Fragrance molecules used in this study.

Name	CAS No.	SMILES
Citronellol	106-22-9	OCCC(CCC=C(C)C)C
Citronellyl formate	105-85-1	O=COCCC(CCC=C(C)C)C
Citronellyl acetate	150-84-5	O=C(OCCC(CCC=C(C)C)C)C
Citronellyl propionate	141-14-0	O=C(OCCC(CCC=C(C)C)C)CC
Citronellyl n-butyrate	141-16-2	O=C(OCCC(CCC=C(C)C)C)CCC
Citronellyl isobutyrate	97-89-2	O=C(OCCC(CCC=C(C)C)C)C(C)C
D-Citronellal	2385-77-5	O=CCC(CCC=C(C)C)C
Citronellyl Nitrile	51556-62-2	C(#N)CC(CCC=C(C)C)C
Geraniol	106-24-1	OCC=C(CCC=C(C)C)C
Tetrahydro geraniol	106-21-8	OCCC(CCCC(C)C)C
Geranyl formate	105-86-2	O=COCC=C(CCC=C(C)C)C
Geranyl acetate	105-87-3	O=C(OCC=C(CCC=C(C)C)C)C
Geranyl propionate	105-90-8	O=C(OCC=C(CCC=C(C)C)C)CC
Geranyl n-butyrate	106-29-6	O=C(OCC=C(CCC=C(C)C)C)CCC
Geranyl isobutyrate	2345-26-8	O=C(OCC=C(CCC=C(C)C)C)C(C)C
Geranyl isovalerate	109-20-6	O=C(OCC=C(CCC=C(C)C)C)CC(C)C
Geranyl nitrile	5146-66-7	CC(=CCCC(=CC#N)C)C
Citral	5392-40-5	O=CC=C(CCC=C(C)C)C

Tetrahydrocitral	5988-91-0	O=C(OC)CCCCCCCC=CC
Nerol	106-25-2	OCC=C(CCC=C(C)C)C
Neryl acetate	141-12-8	O=C(OCC=C(CCC=C(C)C)C)C
Linalool	78-70-6	OC(C=C)(CCC=C(C)C)C
Tetrahydro linalool	78-69-3	OC(CCCC(C)C)(CC)C
Linalyl formate	115-99-1	O=COC(C=C)(CCC=C(C)C)C
Linalyl acetate	115-95-7	O=C(OC(C=C)(CCC=C(C)C)C)C
Linalyl propionate	144-39-8	O=C(OC(C=C)(CCC=C(C)C)C)CC
Linalyl n-butyrate	78-36-4	O=C(OC(C=C)(CCC=C(C)C)C)CCC
Linalyl isobutyrate	78-35-3	O=C(OC(C=C)(CCC=C(C)C)C)C(C)C
Ethyl linalool	10339-55-6	C=CC(O)(CC/C=C(C)/CC)C
Ethyl linalyl acetate	61931-80-4	O=C(OC(C=C)(CCC=C(CC)C)C)C
Ocimene	13877-91-3	C(=CCC=C(C=C)C)(C)C
Muguol	18479-54-4	OC(C=CC=C(C)C)(CC)C
Tetrahydro muguol	78-69-3	OC(CCCC(C)C)(CC)C
Myrcene	123-35-3	C(C=C)(=C)CCC=C(C)C
Myrcenol	543-39-5	OC(CCCC(C=C)=C)(C)C
Dihydro myrcenol	18479-59-9	OC(CCCC(C=C)C)(C)C
Farnesol	4602-84-0	OCC=C(CCC=C(CCC=C(C)C)C)C
Nerolidol	40716-66-3	OC(C=C)(CCC=C(CCC=C(C)C)C)C
D-limonene	5989-27-5	C(=CCC(C(=C)C)C1)(C1)C
Dipentene	138-86-3	C(=CCC(C(=C)C)C1)(C1)C

α-Terpinene	99-86-5	C(=CC=C(C1)C)(C1)C(C)C
α-Terpineol	98-55-5	OC(C(CCC(=C1)C)C1)(C)C
Dihydroterpineo l	498-81-7	OC(C(CCC(C1)C)C1)(C)C
Terpinyl acetate	80-26-2	O=C(OC(C(CCC(=C1)C)C1)(C)C)C
Terpineol	8000-41-7	OC(C1CC=C(CC1)C)(C)C
γ-Terpinene	99-85-4	C(=CCC(=C1)C)(C1)C(C)C
Terpinen-4-ol	562-74-3	OC(CCC(=C1)C)(C1)C(C)C
1,8-Cineol	470-82-6	CC1(C2CCC(O1)(CC2)C)C
Isobornyl acetate	125-12-2	O=C(OC(C(C(C1C2)(C)C)(C2)C)C1)C
α-Pinene	80-56-8	C(C(CC1C2)C1(C)C)(=C2)C
β-Pinene	127-91-3	C(C(CC1C2)C1(C)C)(C2)=C
L-Menthol	2216-51-5	CC1CCC(C(C1)O)C(C)C
Dihydropinyl acetate	80-25-1	C1(C(C)(C)OC(=O)C)CCC(C)CC1
L-Menthone	14073-97-3	CC1CCC(C(=O)C1)C(C)C
iso-menthone	491-07-6	O=C(C(CCC1C)C(C)C)C1
L-Carvone	6485-40-1	CC1=CCC(CC1=O)C(=C)C
β-Caryophyllene	87-44-5	C(=CCCC(C(C(C1(C)C)C2)C1)=C)(C2)C
Caryophyllene acetate	57082-24-3	O=C(OC(C(C(C1(C)C)CCC2(CC3)C)C1)(C2)C3)C
Cedrene	19069-48-8	C(C(C(C1C(=C2)C)(C)C)CC3)(C1)(C3C)C2
Cedryl acetate	77-54-3	O=C(OC(C(CC(C1CC2)(C2C)C3)C1(C)C)(C3)C)C
Benzyl alcohol	100-51-6	OCc(cccc1)c1
Benzyl formate	104-57-4	O=COCc(cccc1)c1
Benzyl acetate	140-11-4	O=C(OCc(cccc1)c1)C

Benzyl Propionate	122-63-4	O=C(OCc(cccc1)c1)CC
Benzyl n-butyrate	103-37-7	O=C(OCc(cccc1)c1)CCC
Benzyl isobutylate	103-28-6	O=C(OCc(cccc1)c1)C(C)C
Benzyl isovalerate	103-38-8	O=C(OCc(cccc1)c1)CC(C)C
Benzyl cinnamate	103-41-3	O=C(OCc(cccc1)c1)C=Cc(cccc2)c2
Benzyl salicylate	118-58-1	O=C(OCc(cccc1)c1)c(c(O)ccc2)c2
Benzyl benzoate	120-51-4	O=C(OCc(cccc1)c1)c(cccc2)c2
Benzaldehyde	100-52-7	O=Cc(cccc1)c1
Ethyl benzoate	93-89-0	O=C(OCC)c(cccc1)c1
Phenethyl alcohol	1960-12-8	OCCc(cccc1)c1
Phenethyl formate	104-62-1	O=COCCc(cccc1)c1
Phenethyl acetate	103-45-7	O=C(OCCc(cccc1)c1)C
Phenethyl Propionate	122-70-3	O=C(OCCc(cccc1)c1)CC
Phenethyl salicylate	87-22-9	O=C(OCCc(cccc1)c1)c(c(O)ccc2)c2
Cinnamic alcohol	104-54-1	OCC=Cc(cccc1)c1
Cinnamic aldehyde	104-55-2	O=CC=Cc(cccc1)c1
Anethole	4180-23-8	O(c(ccc(c1)C=CC)c1)C
Eugenol	97-53-0	O(c(c(O)ccc1CC=C)c1)C

Dihydroeugenol	2785-87-7	O(c(c(O)ccc1CCC)c1)C
Eugenol acetate	93-28-7	O=C(Oc(c(OC)cc(c1)CC=C)c1)C
Iso-eugenol	5932-68-3	O(c(c(O)ccc1C=CC)c1)C
Methyl iso-eugenol	93-16-3	CC=CC1=CC(=C(C=C1)OC)OC
Anise alcohol	105-13-5	O(c(ccc(c1)CO)c1)C
Anisyl acetate	104-21-2	O=C(OCc(ccc(OC)c1)c1)C
Anisaldehyde	123-11-5	O=Cc(ccc(OC)c1)c1
p-Cymene	99-87-6	c(ccc(c1)C)(c1)C(C)C
Thymol	89-83-8	Oc(c(ccc1C)C(C)C)c1
1-pentanol	71-41-0	OCCCCC
isopentyl alcohol	123-51-3	OCCC(C)C
1-Hexanol	111-27-3	OCCCCCC
1-Hexanal	66-25-1	O=CCCCCC
n-Hexanoic acid	142-62-1	CCCCCC(=O)O
Ethyl hexanoate	123-66-0	O=C(OCC)CCCCC
1-Heptanol	111-70-6	OCCCCCCC
1-Heptanal	111-71-7	O=CCCCCCC
Ethyl heptanoate	106-30-9	O=C(OCC)CCCCCC
2-Heptanone	110-43-0	CCCCCC(=O)C
1-Octanol	111-87-5	OCCCCCCCC
1-Octanal	124-13-0	O=CCCCCCCC
n-Octanoic acid	124-07-2	CCCCCCCC(O)=O
Ethyl octanoate	106-32-1	O=C(OCC)CCCCCCC

2-Ocanone	111-13-7	CCCCCCC(=O)C
1-Nonanol	143-08-8	OCCCCCCCCC
1-Nonanal	124-19-6	O=CCCCCCCCC
n-Nonanoic acid	112-05-0	CCCCCCCCC(=O)O
Ethyl nonanoate	123-29-5	O=C(OCC)CCCCCCCC
1-Decanol	112-30-1	OCCCCCCCCCC
1-Decanal	112-31-2	O=CCCCCCCCCC
n-Decanoic acid	334-48-5	CCCCCCCCCC(=O)O
Ethyl decanoate	110-38-3	O=C(OCC)CCCCCCCCC
n-Undecanal	112-44-7	O=CCCCCCCCCCC
n-Undecanoic acid	112-37-8	CCCCCCCCCCC(=O)O
2-Undecanone	112-12-9	CCCCCCCCCC(=O)C
1-Dodecanol	112-53-8	OCCCCCCCCCCCC
1-Dodecanal	112-54-9	O=CCCCCCCCCCCC
2-Methylundecanal	110-41-8	O=CC(CCCCCCCCC)C
p-Cresol	106-44-5	Oc(ccc(c1)C)c1
Dimethyl benzyl carbinol	100-86-7	OC(Cc(cccc1)c1)(C)C
trans-2-Hexenal	6728-26-3	O=CC=CCCC
cis-3-Hexenol	928-96-1	OCCC=CCC
trans-2-Hexenol	928-95-0	OCC=CCCC
trans-2-Hexenyl acetate	2497-18-9	O=C(OCC=CCCC)C

cis-3-Hexenyl formate	33467-73-1	O=COCCC=CCC
Melonal	106-72-9	O=CC(CCC=C(C)C)C
Propylene glycol	57-55-6	OCC(O)C
3-Phenylpropanal	104-53-0	O=CCCc(cccc1)c1
2-Methyl-2-pentanoic acid	3142-72-1	O=C(O)C(=CCC)C
Isononylaldehyde	5435-64-3	O=CCC(CC(C)(C)C)C
Cinnamyl formate	104-65-4	O=COCC=Cc(cccc1)c1
p-Tolualdehyde	104-87-0	O=Cc(ccc(c1)C)c1
Phenethyl alcohol	1960/12/8	OCCc(cccc1)c1
Citral	5392-40-5	O=CC=C(CCC=C(C)C)C
cis-6-Nonenal	2277-19-2	O=CCCCCC=CCC
trans-2-Decenal	3913-81-3	O=CC=CCCCCCCC
trans-3-Hexenol	928-97-2	OCCC=CCC
3-methoxy-3-methylbutanol	56539-66-3	O(C(CCO)(C)C)C
Acetic acid	64-19-7	O=C(O)C
Cyclovertal	67801-65-4	O=CC(C(CC=C1C)C)C1
Isoamil acetate	123-92-2	O=C(OCCC(C)C)C
Isobutyl acetate	110-19-0	O=C(OCC(C)C)C
10-Undecenal	112-45-8	O=CCCCCCCCCC=C
p-Methylanisole	104-93-8	O(c(ccc(c1)C)c1)C
Cis-4-decenal	21662-09-9	O=CCCC=CCCCCC
trans-4-Decenal	65405-70-1	O=CCCC=CCCCCC

Dimetol	13254-34-7	OC(CCCC(C)C)(C)C
Ethyl isovalerate	108-64-5	O=C(OCC)CC(C)C
Hydratropic aldehyde	93-53-8	O=CC(c(cccc1)c1)C
Hydroxycitronellal	107-75-5	O=CCC(CCCC(O)(C)C)C
10-Undecenal	112-45-8	O=CCCCCCCCCC=C
Linalool oxide	60047-17-8	O(C(C=C)(CC1)C)C1C(O)(C)C
7-Methoxycitronellal	3613-30-7	O=CCC(CCCC(OC)(C)C)C
2-Methyl-4-phenyl-2-butanol	103-05-9	OC(CCc(cccc1)c1)(C)C
Prenyl acetate	1191-16-8	O=C(OCC=C(C)C)C
Rose oxide	16409-43-1	O(C(C=C(C)C)CC(C1)C)C1
Rose oxide	16409-43-1	O(C(C=C(C)C)CC(C1)C)C1
3,5,5-Trimethylhexanol	3452-97-9	OCCC(CC(C)(C)C)C
Ethanol	64-17-5	OCC
Dimyrcetol	25279-09-8<br>18479-58-8<br>(mixture 50:50)	O=COC(CCCC(C=C)C)(C)C
Hydratropic alcohol	1123-85-9	OCC(c(cccc1)c1)C
Hydroxycitronellal	107-75-5	O=CCC(CCCC(O)(C)C)C
trans-2-Undecanal	53448-07-0	O=CC=CCCCCCCCC
n-Butyl acetate	123-86-4	O=C(OCCCC)C
syringa aldehyde	104-09-6	O=CCc(ccc(c1)C)c1

cis-6-Nonenol	35854-86-5	OCCCCCC=CCC
Sulfurol	137-00-8	n(c(c(s1)CCO)C)c1
Lymolene	18479-58-8	OC(CCCC(C=C)C)(C)C
2-methylbutyric acid	116-53-0	O=C(O)C(CC)C
Methyl isovalerate	556-24-1	O=C(OC)CC(C)C
γ-Valerolactone	108-29-2	O=C(OC(C1)C)C1
2,3,5-Trimethylpyrazine	14667-55-1	n(c(c(nc1C)C)C)c1

Table S2. The Performance of the GPRs with $k_{0.5}$ or $k_{2.5}$ kernel functions using weighted means as statistical descriptors.

	GPRs					
Kernel	$k_{0.5}$	$k_{0.5}$	$k_{0.5}$	$k_{2.5}$	$k_{2.5}$	$k_{2.5}$
Transform	-	log	logit	-	log	logit
$\mathbf{R^2_{cv}}$	0.71	0.56	0.69	0.71	0.52	0.67
$\mathbf{RMSE_{cv}}$	16.1	19.8	16.0	16.2	20.7	16.5
$\mathbf{MAE_{cv}}$	11.1	12.1	10.1	11.4	12.4	10.3
$\mathbf{MAPE_{cv}}$	241	84.4	89.8	255	87.1	92.9

Table S3. The performance of the GPRs or $k_{0.5}$ and $k_{2.5}$ kernel functions using weighted means and weighted standard deviations as statistical descriptors.

	GPRs					
Kernel	$k_{0.5}$	$k_{0.5}$	$k_{0.5}$	$k_{2.5}$	$k_{2.5}$	$k_{2.5}$
Transform	-	log	logit	-	log	logit
$\mathbf{R^2_{cv}}$	0.72	0.58	0.70	0.72	0.59	0.71
$\mathbf{RMSE_{cv}}$	15.9	19.4	15.6	15.8	19.1	15.6
$\mathbf{MAE_{cv}}$	11.0	11.8	9.78	11.0	11.7	9.75
$\mathbf{MAPE_{cv}}$	227	81.2	86.3	237	85.3	90.6

Table S4. The performance of the GPRs with $k_{0.5}$ or $k_{2.5}$ kernel functions using weighted mean, weighted standard deviation, and maximum and minimum values as statistical descriptors.

	GPRs					
Kernel	$k_{0.5}$	$k_{0.5}$	$k_{0.5}$	$k_{2.5}$	$k_{2.5}$	$k_{2.5}$
Transform	-	log	logit	-	log	logit
$\mathbf{R^2_{cv}}$	0.72	0.61	0.71	0.72	0.57	0.71
$\mathbf{RMSE_{cv}}$	15.8	18.8	15.4	15.8	19.7	15.4
$\mathbf{MAE_{cv}}$	10.9	11.5	9.67	10.9	11.7	9.62
$\mathbf{MAPE_{cv}}$	212	79.6	84.4	225	88.2	90.2
